# Regioselective palladium-catalysed aerobic oxidation of dextran and its use as a bio-based binder in paperboard coatings†

**DOI:** 10.1039/d3gc04204a

**Published:** 2024-02-23

**Authors:** Sarina C. Maßmann, Gerald A. Metselaar, Derk Jan van Dijken, Keimpe J. van den Berg, Martin D. Witte, Adriaan J. Minnaard

**Affiliations:** a Stratingh Institute for Chemistry, University of Groningen Nijenborgh 7 9747 AG Groningen The Netherlands a.j.minnaard@rug.nl; b BASF Nederland BV Innovatielaan 1 Heerenveen 8447 SN The Netherlands; c Akzo Nobel Car Refinishes BV Rijksstraatweg 31 Sassenheim 2171 AJ The Netherlands

## Abstract

The coatings industry is aiming to replace petrochemical-based binders in products such as paints and lacquers with bio-based alternatives. Native polysaccharide additives are already used, especially as adhesives, and here we show the use of oxidised dextran as a bio-based binder additive. Linear dextran with a molecular weight of 6 kDa was aerobically oxidised in water at the C3-position of its glucose units, catalysed by [(neocuproine)PdOAc]_2_(OTf)_2_. The resulting keto-dextran with different oxidation degrees was studied using adipic dihydrazide as a crosslinker in combination with the commercial petrochemical-based binder Joncryl®. Coating experiments show that part of the Joncryl® can be replaced by keto-dextran while maintaining the desired performance.

## Abstract 2

Dextran can be selectively oxidized at the C3-position and subsequently crosslinked with dihydrazides.

## Graphical abstract

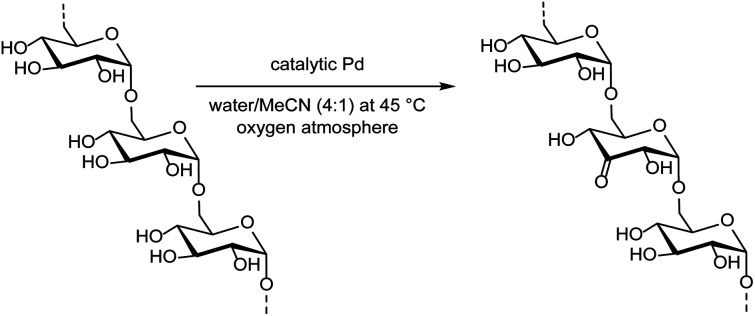

## Introduction

The transition from petrochemical-based to bio-based products is a big challenge for the coatings industry.^1^ Dependency on fossil fuels and oil-rich countries is one side of the problem, but also the fact that most commercial plastics, paints, and lacquers are non-degradable is an important issue.^2^

Inks, paints, and lacquers can be summarised under the term ‘coatings’. Coatings are barriers with protective properties, *e.g.* anti-corrosion, water or/and solvent resistance, as well as weather protection, and are adhered to materials to enhance their lifetime.^3^ In a coating, the binder is a solution or dispersion of a polymer or resin in the solvent or diluent which provides the basis for a coating film, often together with a crosslinker.^4^ Next to the binder and the crosslinker additives such as catalysts, thickeners, flow agents, or surfactants can influence the film-formation process or prevent foaming. Pigments provide colour, opacity, and other optical effects. These are less used to influence the physical properties of the coating but more because of aesthetic reasons although some pigments may act as anti-corrosive or heat protection agents.

Lastly, extenders can be added for several other purposes often related to the primary.^4^

In the last two decades, the polymer industry established waterborne polymer dispersions as an alternative to organic solvent-borne binders, reducing not only the amount of volatile organic compounds (VOCs) being emitted into the atmosphere,^5^ but also giving a foundation for water soluble bio-based additives. The binders commonly are based on a copolymer of polystyrene (PS) and poly(vinyl) acrylics.^6^ The acrylics part of the polymer can be functionalised (*e.g.* with an epoxide or a ketone) to make these suitable for crosslinking agents for waterborne coatings such as dihydrazides.^7^ The Schiff base formation between hydrazides and carbonyl moieties results in hydrazones and takes place rather slowly. However, the formation of hydrazones at ambient temperatures is well-known in literature for several coating applications.^8–14^ This is because the hydrazone formation is a dehydration reaction whose equilibrium is on the side of the hydrazone and accelerated during the drying process of the coating. The most common dihydrazide crosslinker is adipic dihydrazide (ADH), and the production of bio-based adipic acid in high yield and selectivity has been developed.^15^ ADH is water soluble, odourless, and has a low toxicity,^16^ which makes it a suitable crosslinker for modern low VOC waterborne coatings that are environmentally friendly and safe to use.^17,18^

In order to make existing waterborne coatings more sustainable or ultimately completely bio-based, the coatings industry aims to replace the aforementioned petrochemical-based binders with bio-based alternatives.

Many abundant bio-materials are composed of polysaccharides.^19^ Polysaccharides have the advantage that their monosaccharide units are connected by strong glycosylic linkages and possess a high number of hydroxyl functionalities. Because of this high number of hydroxyl groups in polysaccharides, the viscosity of their aqueous solutions is rather high.^20^ In order to use polysaccharide solutions as bio-based additive, the rheological behaviour of aqueous polysaccharide solutions must match with the rheology of common commercial binders in waterborne coatings.^21^ Commercial binders are often supplied as an aqueous dispersion with a solid content of 40–60% (solids of polymeric resin binder in water),^22^ but often lower solid contents of polysaccharide binders (∼10 wt%) are used because of their viscosity.^23–25^ Without further functionalization, the polysaccharide starch/dextrin is already being used *e.g.* as filler,^5^ as a surfactant,^26–28^ in higher concentrations as a thickener,^29–31^ or dyed with a pigment.^32^

In order to make use of polysaccharides as (co-)binders in combination with ADH as a crosslinker, crosslinking handles need to be installed. Therefore the polysaccharide starting material is oxidised to generate carbonyl moieties that can react with the hydrazide. However, controlled modifications such as oxidations are difficult, due to the similar reactivity of the hydroxyl groups.

The modification of carbohydrates using transition metal-catalyzed reactions is an important research field, both in heterogeneous catalysis and in enzyme catalysis.^33,34^ Efforts focus on biomass conversion, and in particular the production of building blocks, so-called platform chemicals, for the chemical industry has seen considerable progress.^35,36^ In these processes, polysaccharides such as (hemi)cellulose, starch and chitin are depolymerized to their monomers that subsequently are dehydrated. A series of transition metal-catalysts is used but palladium is a prominent member.^37^ Also the polymers themselves, in particular cellulose and starch,^38,39^ are modified to improve their performance as materials.

**Fig. 1:**
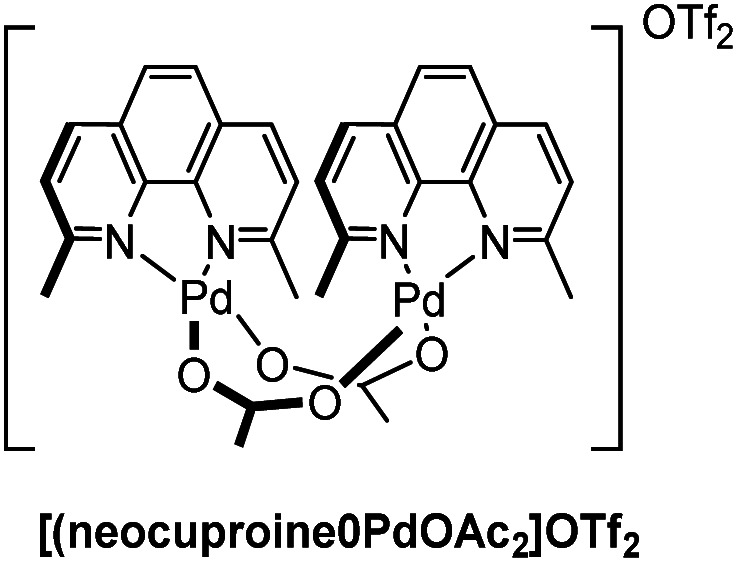
Molecular structure of the Pd catalyst.

Our group demonstrated the site-selective oxidation of α-methyl-d-glucopyranoside at the C3 position using a homogeneous monocationic 2,9-dimethyl phenanthroline (neocuproine) palladium complex (Fig. 1),^40^ introduced by Waymouth *et al.*^41^ We also showed the regioselective oxidation of β-methyl-d-maltoside and β-methyl-d-cellobioside at this position in the terminal residue with the same palladium catalyst, benzoquinone (BQ) as terminal oxidant, and dioxane/DMSO (4 : 1) as solvent.^40^ The substrate scope was extended to the oligomer β-d-maltoheptaosyl azide and its application in chemical biology was demonstrated.^42–45^

The catalyst chelates to vicinal hydroxyl groups and oxidizes the most activated one. Due to the (1,4)-linkages in oligomaltoses, chelation of the C3/C4–OH is not possible and chelation of the C2/C3–OH suffers from steric hindrance because of the catalyst's bulky neocuproine ligand. For this reason only the C3–OH of the terminal residue is available for oxidation in (1,4)-linked saccharides,^42^*e.g.* cellulose, starch and chitin.

**Fig. 2:**
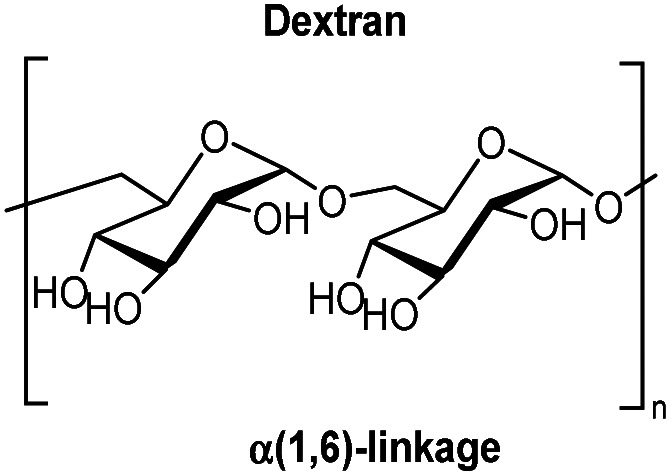
The molecular structure of dextran.

Dextran, on the other hand, is an α(1,6)-linked glucan (Fig. 2) produced from sucrose by the bacterial extracellular enzyme dextransucrase found in Leuconostocci, Gluconobacter, Streptococci, and Lactobacilli.^46–53^ This is a potential starting material for a binder, provided that part of the C3 hydroxy groups can be oxidized to the corresponding ketones.

At lower degrees of polymerisation, dextran is linear. At higher molecular weight, however, dextran shows some degree of branching at the C3- and C4-position,^54,55^ which limits the number of available positions for oxidation (see ESI†).

The oxidation of dextran by NaIO_4_ is well-known,^56^ and already used for several applications, especially in medicine.^57–62^ Periodate-mediated oxidation of vicinal hydroxyl groups in glucose units forms two aldehyde moieties which rapidly react with hydrazides, but are close to each other and this can lead to the formation of substituted dioxanes.^63^ As a result, there is only partial control over the number of available crosslinking points in periodate-oxidized dextran. The potential advantage of keto-dextran as a bio-based binder compared to periodate-oxidized dextran is the control over the number of ketone functions and their lower reactivity.

Although a direct functionalization or even crosslinking on the secondary hydroxyl groups within (linear) dextran is possible and already used in medicinal applications,^64,65^ it is less suitable for co-crosslinking with (petrol-based) binders because it is due to the similar reactivity of those hydroxyl groups less controlled.

Here we demonstrate the palladium catalysed aerobic oxidation of linear dextran. The use of oxygen instead of an organic oxidant such as benzoquinone, as reported earlier for mono- and oligosaccharides,^66^ makes purification by column chromatography redundant. Treatment with active carbon and filtration over Celite on a glass fibre filter is sufficient to remove the palladium catalyst (see ESI†). The degree of oxidation of the keto-dextran, defined as the number of C3-keto groups divided by the total number of C3-positions in the polymer, can be varied from 0–30% mainly by increasing the catalyst loading. Mixtures of these functionalised dextrans with a commercial keto-functionalised Joncryl® binder and ADH as a crosslinker have been applied as bar-coatings on an unsealed Leneta paperboard test card. It is shown that part of the Joncryl® binder can be replaced by oxidized dextran.

## Results and discussion

**Fig. 3:**
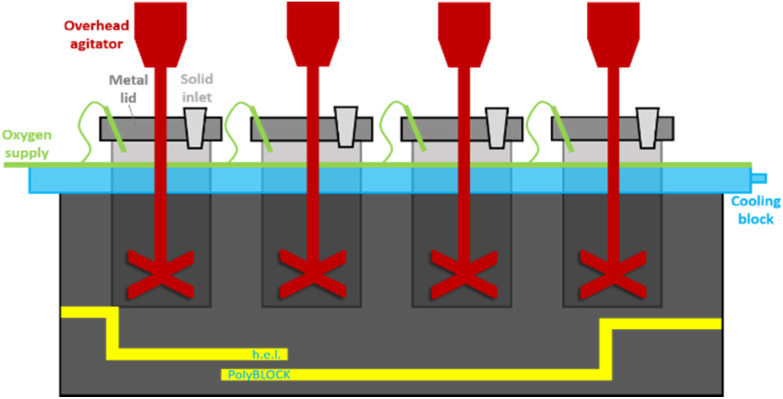
Schematic set-up of the PolyBLOCK® reactor with four glass vessels of 200 mL, a direct oxygen supply, overhead stirring, and a cooling block with water supply.

The overarching goal of this research was the production of a well-performing coating on paperboard with the highest amount of dextran possible. In order to make linear dextran a crosslinkable bio-based building block for waterborne coatings, it was oxidised by palladium neocuproine triflate and oxygen as terminal oxidant. For the aerobic oxidation experiments of dextran we used an automated synthesis platform (PolyBLOCK®). The advantages are a high reproducibility and a full continuous control by the software labCONSOL®. Moreover, overhead agitation was readily installed as well as a direct oxygen supply (Fig. 3).

**Fig. 4:**
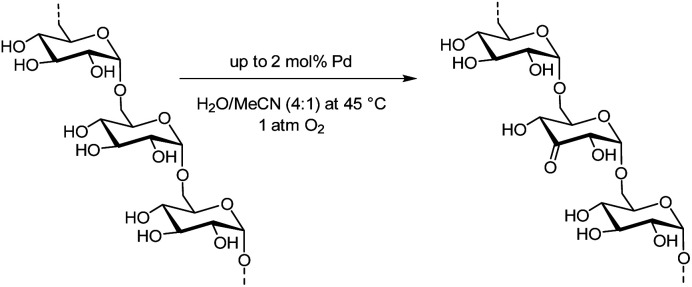
Regioselective aerobic oxidation of linear dextran.

The PolyBLOCK® was equipped with two to four glass vessels (200 mL) in parallel. A low reaction volume (25 mL) was used to allow a high mass transfer from the gas phase to the liquid phase.^67^ By varying the catalyst loading, linear low molecular weight keto-dextran was synthesized with varying oxidation degrees (5%, 10%, 17%, 21%, 27%, and 37%). The percentage refers to the number of residues. Thus, an oxidation degree of 30% means that 30% of the residues has been oxidized. For dextran with an MW of 6 kDa this corresponds to 12 residues out of 37 residues on average and corresponds to an approximate TON of the palladium catalyst of 80. The distribution of the oxidized residues over the chains was studied later (*vide infra*). The palladium and the ligand could be recovered separately by filtration after the reaction, but the catalyst was not re-used. Recovery of the oxidized dextran was high but could not be determined accurately as it was difficult to remove residual water from the product (Fig. 4).

With an increased polymer weight, the analysis of the reaction mixture by quantitative NMR became more difficult due to broadening of the signals. Although integration of the signals was possible, the analysis was less accurate (for the use of qNMR, see ESI†).

**Fig. 5:**
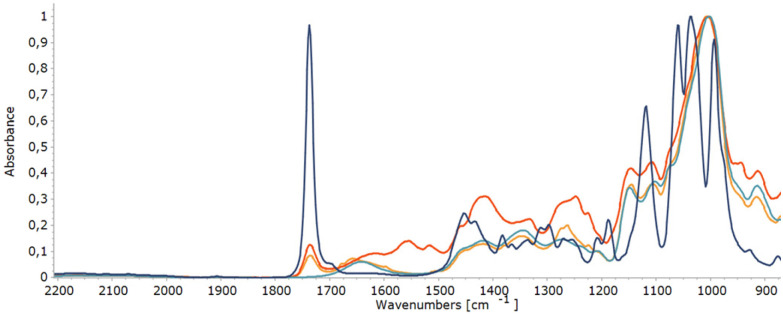
IR spectra of solid linear dextran (green), linear keto-dextran 10% oxidation degree (yellow), linear keto-dextran 17% oxidation degree (red), and 3-keto-α-methyl-d-glucopyranoside (blue). The *Y*-axis is normalized for the absorption at 1000 cm^−1^.

Carbonyl groups absorb strongly in the infrared, between 1760 cm^−1^ and 1665 cm^−1^, because of the stretching vibration of the C=O bond.^68^ In search for a complementary analysis method to monitor the oxidation degree during site-selective oxidation of dextran, the suitability of infrared spectroscopy (IR) was assessed. Gratifyingly, the C=O stretch vibration of the carbonyl moiety in methyl 3-keto-glucoside as the reference model, as well as in oxidised dextran, is readily observed at 1735 cm^−1^ (Fig. 5). Determination of the oxidation degree using the area under this absorption in the IR spectrum was in line with the results of the analysis performed by qNMR (see ESI, Table 2†).

**Fig. 6:**
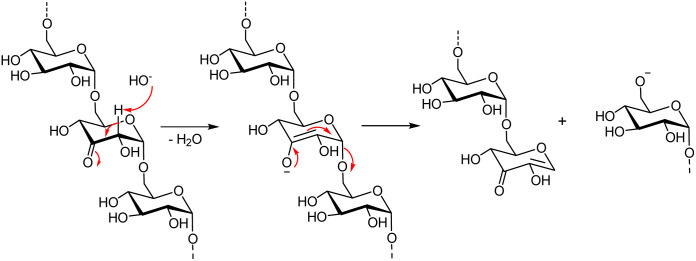
Base-catalysed E1bc of keto-dextran.

In order to investigate the distribution of the carbonyl groups in the linear keto-dextran with an oxidation degree of 10%, we carried out a fragmentation of the polysaccharide chain with a base-catalysed E1cb-type elimination reaction. Potassium carbonate was selected as a base, producing a low concentration of hydroxide which abstracts the α-acidic C2-proton adjacent to the oxidised C3-position thereby forming an enolate (enediolate) (Fig. 6). E1cb elimination causes cleavage of the glycosylic bond of the oxidised glucose unit. The fragmentation, therefore, happens exclusively at the anomeric center of the oxidized glucose residues. Hence, the size of the resulting fragments gives an indication of the distribution of the carbonyl groups within the keto-dextran chain.

In order to analyse the length-distribution of the fragments, the reaction mixture was separated by cut-off centrifugal filters (5 kDa, 3 kDa, and 1 kDa). The filtrate was lyophilized, weighed and subsequently analysed by qNMR (see ESI†). The fragmentation experiments showed that the oxidised glucose residues were evenly distributed. This can be concluded because the fragments of a well-distributed oxidized dextran should all have a molecular weight of ∼600 Da and the fragments were indeed <1 kDa. The uniform distribution is versatile because it means that subsequent crosslinking of the carbonyl groups with a dihydrazide is uniformly distributed too, forming a well-defined crosslinking network and therefore coating film.

The crosslinking ability of the keto-dextrans was studied by mixing with ADH in water and applying droplet coatings on a glass microscopy plate. The droplet coatings were dried at r.t. for 24 h. Then the water resistance was tested by applying a water stream of 10 mL over the droplet coatings. This showed that the higher the degree of oxidation, and therefore crosslinking, the better the water resistance (details see ESI†). However, when leaving 1 mL of water for 10 min on the droplet coatings even the ones with a higher degree of oxidation showed swelling.

The keto-functionalised Joncryl® binder is a self-crosslinking acrylic emulsion with ADH already added. It is a binder for water-based inks used for surface printing on film substrates. In order to analyse the co-crosslinking behaviour with the oxidised dextran, the binder was in this case prepared without the addition of ADH, and the latter was added later in the desired amounts. The commercial binder consists of 5% diacetone acrylamide (DAAM) as part of a copolymer which, hence, contains ketones as functionality. To reduce the amount of the Joncryl® copolymer in the binder, part of it was replaced by either dextran or keto-dextran. The amount was varied between 0–25 wt% to determine how much bio-based additive can be added before a loss in performance occurs. The performance was defined by the positive aspects *i.e.* no gelation, high water resistance and high amount of bio-polymer added. Non-oxidised dextran was taken as reference material. As the functionalised polysaccharide keto-dextran with varying degrees of oxidation (*vide supra*) was used. The degrees of oxidation of keto-dextran were 10%, 21%, and 27%. The commercial binder and the bio-polymer were blended with water and an aqueous 0.43 M ADH solution to form a new binder dispersion.

**Fig. 7:**
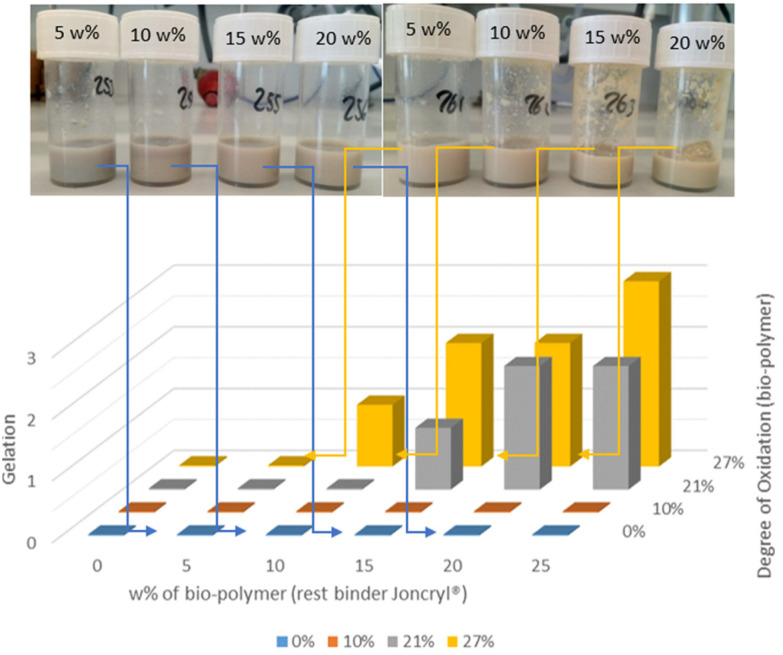
The degree of gelation depending on the degree of oxidation of the bio-polymer and its amount added.

**Fig. 8:**
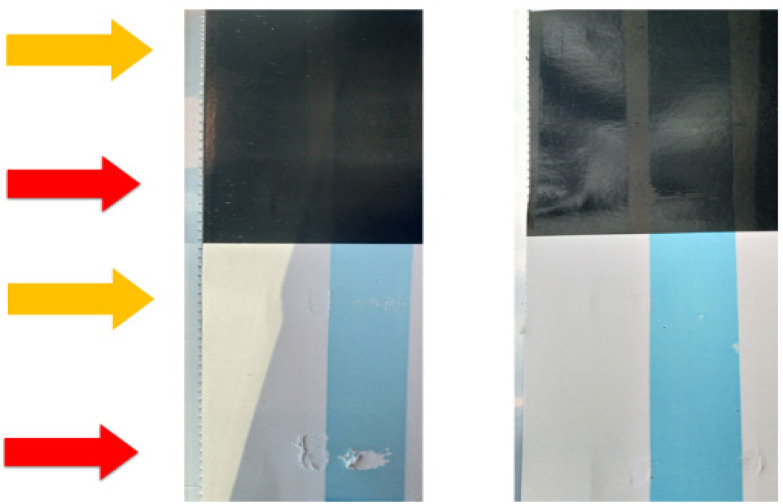
Bar-coating of Joncryl® binder with 20 wt% bio-polymer and ADH. Final results of water resistance tests. Bio-polymer left: dextran (6 kDa) and right: keto-dextran with an oxidation degree of 10%. Orange arrows showing short-time water resistance tests; red arrows showing long-term water resistance tests (see also ESI†).

Upon mixing Joncryl® with keto-dextran and ADH, several of the blends showed immediate gelation, evident from the formation of a precipitate on the inside wall of the vial (Fig. 7, right). It was not possible to redissolve the formed solids by adding more water. Therefore, keto-dextran with an oxidation degree of 37% was not used. Gelation was not observed in the mixtures composed of the commercial binder with ADH and unfunctionalised (native) dextran (Fig. 7, left). The instantaneous gelation is an indication that excessive crosslinking, that is hydrazone formation, already occurs in solution before drying of the coating. Gelation is an undesired process since it hampers the use of the mixture in the coating application process. To visually represent the gelation of the various Joncryl®–keto-dextran mixtures, the extent of gelation was scored on a scale of zero to three, where three means extensive gelation-caused precipitation and zero means no gelation. The qualitative scores for the various mixtures are plotted in a bar diagram (Fig. 8).

**Fig. 9:**
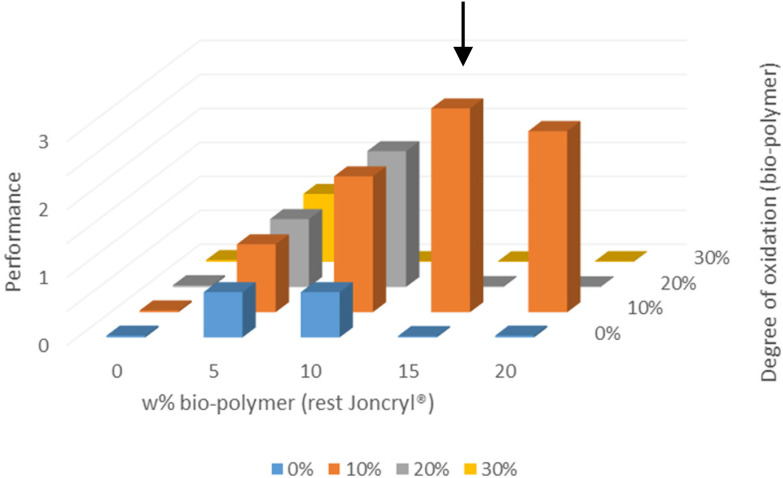
Performance of coatings depending on the degree of oxidation of the bio-polymer and its amount added. The properties of the coatings were scored qualitatively from poor to very good. Positive aspects received the highest scores. The overall performance was obtained by multiplying the different scores and normalizing them to 3.

From the bar diagram, it is readily concluded that the gelation score increases with the degree of oxidation and with the amount of biopolymer weight percentage. This is especially noticeable for the mixtures containing keto-dextran with an oxidation degree of 27% (yellow bars). All the blends containing keto-dextran with 27% oxidation, showed some gelation except for the sample that contained only 5 wt%. The samples formed with keto-dextran with a degree of oxidation of 10%, as well as the reference mixture of the commercial binder with ADH and native dextran did not show any visible gelation. These results suggest that there is a critical concentration of ketone-moieties for intermolecular crosslinking of keto-dextran with ADH happening in solution. The remaining blends that did not show gelation during the mixing process were used with and without a blue pigment (Fig. 9) for bar-coating tests on unsealed paper (drying 1 min at 60 °C and 24 h at r.t.). In order to investigate how the added amount of bio-polymer, as well as its degree of functionalisation affected the water resistance of the coatings, water droplets (∼100 μL) were applied to the top section of the lack and white area of the coated test card (Fig. 8, orange arrow).

After 1 min, the area where the water droplets had been applied was rubbed 30 times with a wet piece of felt according to the industrial standard. The gloss was examined on the black part and a possible removal of the blue colour on the white part of the test card. Furthermore, the bottom section of both test areas of the card was soaked with a wet towel (Fig. 8, red arrow), which was removed after 60 min.

Subsequently, the water resistance was tested again by rubbing to evaluate the long-lasting resistance of the coatings against water. Fig. 8 shows the coatings containing 20 wt% bio-polymer and stoichiometric amounts of ADH. There is a clear difference between the coatings containing the native, unfunctionalised dextran (a) and those containing keto-dextran with an oxidation degree of 10% (b). Coating (b) did not lose its gloss (black area) and the colour was still intact (white area) which both indicate good water resistance because there was almost no coating removed by the rubbing. The coatings with unmodified bio-polymers, on the other hand, did show removal of the coating after rubbing and the same was observed for the reference coatings where no ADH was added as crosslinker (see ESI†). Comparing the long-time water resistance of all bar-coatings, it was possible to replace a part of the Joncryl® binder by oxidised dextran. As this is not the case for native dextran, this shows that the keto-dextran most probably forms (co)crosslinks in the presence of ADH, thereby producing a water-resistant coating film. The use of the oxidized dextran as blend partner, however, is hampered by gelation which occurs at higher degrees of oxidation, and larger amounts of keto-dextran added. To visually represent the overall performance of the coatings of the various Joncryl®–keto-dextran mixtures, the blends were given a score for their gelation behaviour, their water resistance in the bar coating test, and the amount of (oxidized) dextran. Positive aspects (*i.e.* no gelation, high water resistance and high amount of bio-polymer) were rated a high number. The overall score of the blend was calculated by multiplying the level of gelation with the level of water resistance of the coatings as well as the added amount of the bio-polymer. It was normalized to a range of 0 to 3, where 0 is a non-existing and 3 is a very good performance (Fig. 9).

Several well-performing coating combinations can be defined. The best-performing coating was found to consist of mixtures using keto-dextran with an oxidation degree of 10% (Fig. 9, black arrow) because no gelation occurred and the coating was still water-resistant despite a high amount of added bio-polymer (15–20 wt%).

## Conclusions

Palladium-catalysed aerobic oxidation of dextran leads to a functionalised dextran in which part of the C3–OH groups have been oxidized. The keto functions are evenly distributed over the polymer.

To reduce the amount of petrochemical-based binder in coatings, native dextran and keto-dextran with different degrees of oxidation were blended with the commercial keto-functionalised Joncryl® binder and adipic dihydrazide as crosslinker. Bar-coatings were formed of the resulting blends on an uncoated paperboard test card and the effect of the bio-polymer on water resistance was assessed.

The short- and long-term water resistance studies revealed that up to 15 wt% of modified dextran with an oxidation degree of 10% could be added to the commercial Joncryl® binder without a loss in performance. Adding more than 15 wt% resulted in decreased water resistance. However, compared to the use of native dextran, the coatings were still more water resistant. The use of 15 wt% of keto-dextran with a higher degree of oxidation led to gelation upon mixing. The gelation-caused precipitate could not be redissolved by adding more water. Nevertheless, it was possible to add 10 wt% of keto-dextran with an oxidation degree of 21% and 5 wt% of keto-dextran with an oxidation degree of 27% without gelation and the resulting coatings had a good water resistance. If the problem of gelation can be attenuated, keto-dextran could be added in higher quantities and perhaps still produce well-performing coatings. The current study shows that the addition of bio-polymers does not necessarily lead to a loss in performance of the coatings. However, further investigations, such as durability, resilience, flexibility, and toughness are necessary in order to make the application of commercial value. In addition, selective oxidation of dextran at the C3 position is probably not reserved for (homogeneous) palladium catalysis, as recently we showed that electrochemical oxidation of glucose is also C3-selective.^69^

## Experimental section

### Materials

Water was purified by a Merck Millipore Direct Water Purification System. MeCN (HPLC grade) was purchased from Boom. Acetic acid (glacial, ReagentPlus®), linear dextran from *Leuconostoc* spp. Merck (lot number BCCF2262), and Celite (Celite® 454), as well as, activated carbon were purchased from Merck and used without further purification. Adipic dihydrazide was provided by BASF. The palladium catalyst [(neocuproine)PdOAc]_2_(OTf_2_) was prepared according to the literature procedure.^70^ Pd(OAc)_2_ was purchased from STREM.

### Aerobic oxidation of dextran

The starting material was dissolved in H_2_O/MeCN (4 : 1). Subsequently, the catalyst was added as a solid (in the reported amounts, see ESI†). An oxygen atmosphere was applied by attaching an oxygen cylinder to the system. After a reaction time of 20–48 h at 45 °C, active carbon was added to the reaction mixture, which was then filtered over Celite and 5 layers of Whatman glass fibre filters (420 μm thick, 0.7 μm pore size). The filtrate was lyophilised.

### Product characterisation using qNMR spectroscopy

NMR spectra were recorded on a Varian AMX600 spectrometer (600 MHz) using D_2_O as solvent. Chemical shift values (*δ* in ppm) are relative to the resonance of the deuterated solvent as the internal standard (D_2_O, *δ* 4.80 ppm for ^1^H). Data are reported as follows: chemical shifts (*δ*), multiplicity (s = singlet, d = doublet, t = triplet, q = quartet, p = pentet, m = multiplet, dd = doublet of doublets, ddd = doublet of doublet of doublets, td = triplet of doublets, qd = quartet of doublets, pd = pentet of doublets), coupling constants *J* (Hz) and integration (MestroNova software). qNMR was performed with 16 scans and a relaxation time of 60 s per scan.

### Product characterisation using FTIR spectroscopy

Fourier transform infrared (FTIR) spectra of lyophilized samples and solid coating films were recorded using a PerkinElmer Spectrum 400 FTIR spectrometer equipped with an ATR unit. The signal intensity is given as absorbance. Calibration was done using 3-keto-α-methyl-d-glucopyranoside (100% oxidation degree) as well as native α-methyl-d-glucopyranoside and 6 kDa dextran (0% oxidation degree).

### Analysis of the distribution of keto-groups in oxidised dextran

To a solution of 6 kDa linear keto-dextran (400 mg, 0.067 mmol) with an oxidation degree of 10%, K_2_CO_3_ (90 mg, 0.65 mmol, 9.8 equiv.) was added. The reaction mixture was heated to 40 °C and stirred overnight. Subsequently, the solution was filtered by cut-off centrifugal filters (Amicon® Ultra-0.5 Centrifugal Filter for 5 kDa, 3 kDa, and 1 kDa). The filtrates were lyophilized, weighted and analyzed by qNMR in D_2_O as solvent.

### Application of bar-coatings

A paint applicator from RK Print-coat Instruments Ltd. England was used. ∼1.5 mL of the clean mixture as well as the pigmented mixture were pipetted next to each other on the (unsealed N2A) Leneta test card. The bar speed was set to 8. The chosen film thickness was 12 μm. The coatings were directly dried at 60 °C for 1 min and then at r.t. for 24 h. The wire wound bar was cleaned after every run with tap water and a clean brush.

### Analysis of water resistance of the bar-coatings

After drying of the coatings, 4 water droplets (∼100 μL) were pipetted on each test chart (one per section). After 1 min, a wet piece of felt was taken and rubbed 30 times on the soaked areas. Then the spots were examined on their colour and gloss (by eye) to evaluate the water resistance. Subsequently, a water-soaked towel was put on each section for 60 min in order to test the long-term water resistance. Afterwards, a wet piece of felt was taken again and rubbed 30 times on the soaked areas.

## Author contributions

All authors took part in the conceptualization of this study. S. C. M. carried out the experimental work, G. A. M., D. J. v. D. and K. J. v. d. B. provided expert advice and support, S. C. M., M. D. W. and A. J. M. wrote the original draft of the manuscript. All authors contributed to the final version of the manuscript.

## Conflicts of interest

There are no conflicts to declare.

## Supplementary Material

GC-026-D3GC04204A-s001
