# Supplementary material for: Regioselective palladium-catalysed aerobic oxidation of dextran and its use as a bio-based binder in paperboard coatings
Source: Green Chem. 2024 Feb 23;26(7):4005–12. doi: 10.1039/d3gc04204a (PMC10986772; doi:10.1039/d3gc04204a)
Supplement: GC-026-D3GC04204A-s001 [file GC-026-D3GC04204A-s001.pdf]

Electronic Supplementary Material (ESI) for Green Chemistry.
This journal is © The Royal Society of Chemistry 2024

Supporting Information

# Regioselective palladium-catalysed aerobic oxidation of dextran and its use as bio-based binder in paperboard coatings

Sarina C. Maßmann,[a] Gerald A. Metselaar,[b] Derk Jan van Dijken,[b] Keimpe J. van den Berg,[c] Martin D. Witte,*,[a] and Adriaan J. Minnaard.*,[a]

## Contents

**1 Structure of linear dextran with a MW of 6 kDa *versus* branched dextran with a MW of 70 kDa**

**2 Oxidation of dextran (6 kDa and 70 kDa)**

**3 NMR and FTIR spectra of (keto-)dextran**

**4 Fragmentation studies of keto-dextran**

**5 Droplet coatings of keto-dextrans with ADH and their analysis by IR spectroscopy**

**6 Bar coatings of keto-dextrans with a commercial keto-functionalised Joncryl® binder and ADH**

**7 Photo evidence of gelation**

**8 Water resistance tests with photo evidence**

**9 References**

*a Stratingh Institute for Chemistry, University of Groningen, Nijenborgh 7, 9747 AG Groningen, The Netherlands, *email: a.j.minnaard@rug.nl*
*b BASF Nederland BV, Innovatielaan 1, Heerenveen, 8447 SN, The Netherlands.*
*c Akzo Nobel Car Refinishes BV, Rijksstraatweg 31, Sassenheim, 2171 AJ, The Netherlands.*

## 1 Structure of linear dextran with a MW of 6 kDa *versus* branched dextran with a MW of 70 kDa

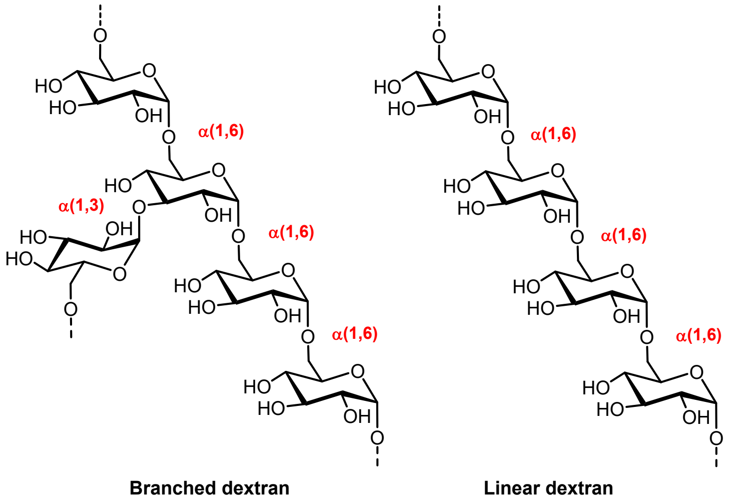


**Fig. 1 Molecular structure of linear low molecular weight dextran and branched high molecular weight dextran.**

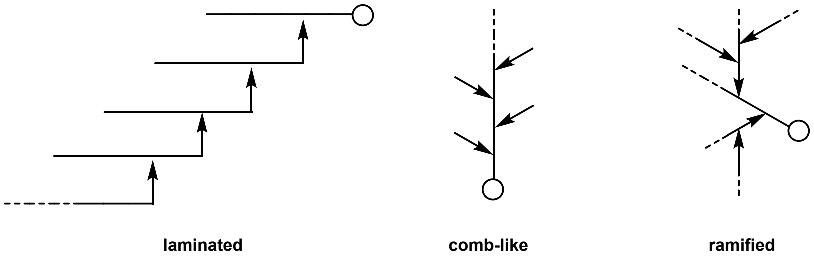


**Fig. 2 Schematic structure representation of possible types of branched dextran secondary structures in water with O terminal, reducing end; — α(1,6)-chain; → α(1,3)-branching; shorter branched chains omitted. Adapted from Sidebotham *et al*.[1]**

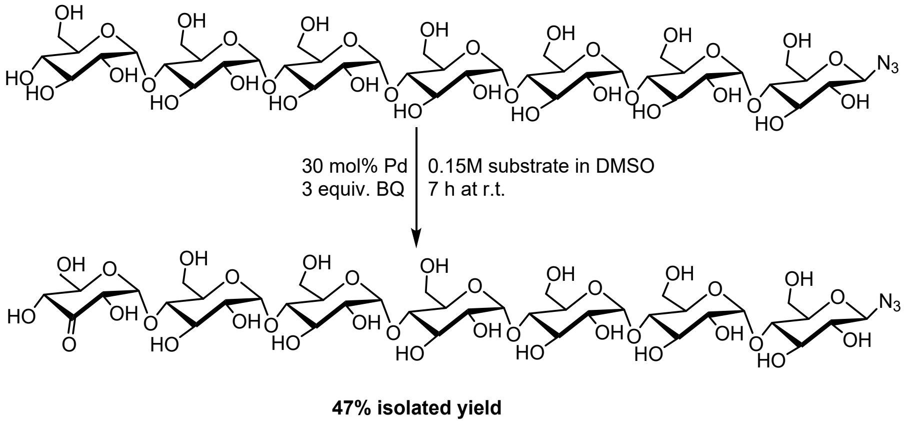


**Fig. 3 Regioselective oxidation of β-D-maltoheptaosyl azide with palladium neocuproine as catalyst. Adapted from Minnaard *et al.*[2]**

## 2 Oxidation of dextran (6 kDa and 70 kDa)

The aerobic oxidation of dextran with a varying molecular weight (lot number of 6 kDa dextran: BCCF2262, and of 70 kDa dextran: BCCD3043 from *Merck*) was carried out in 200 mL glass vessels of a H.E.L. PolyBLOCK® reactor. The reactor is a fully automated synthesis platform (Fig. 3) with overhead agitation as well as an oxygen line.

Oxidation is reported in % of the number of residues. Thus, an oxidation degree of 30% means that 30% of the residues has been oxidized. For dextran with an MW of 6 kDa this corresponds to 12 residues out of 37 residues on average. For this reaction this means an approximate TON of the palladium catalyst of 80. The palladium was recovered by filtration after the reaction but the catalyst was not re-used. Recovery of the dextran was high but could not be determined accurately as it was difficult to remove residual water from the product.

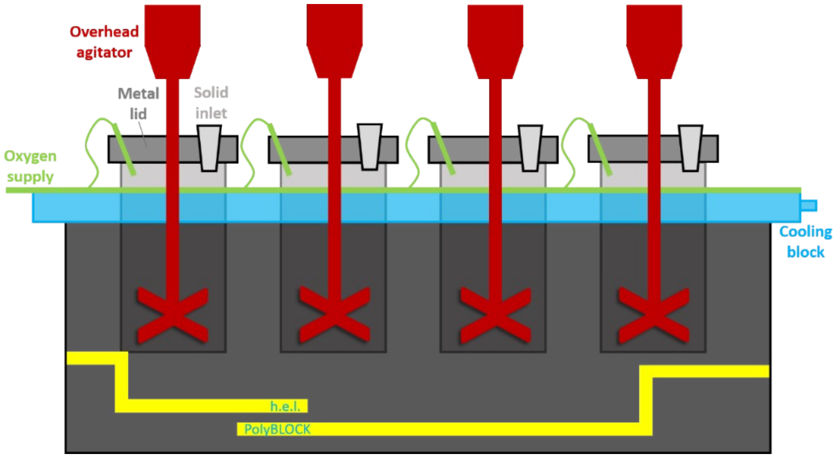


**Fig. 4 Schematic set-up plan of PolyBLOCK® reactor with 4 glass vessels of 200 mL, a direct oxygen supply, overhead stirring, and a cooling block with water supply.**

The starting material was dissolved in $H_2O$/MeCN (in the reported ratio). Afterwards, the catalyst was added as a solid. An oxygen atmosphere was applied by attaching an oxygen cylinder to the system. After a reaction time of 20 h at 45 °C, charcoal was added to the reaction mixture, and the mixture was then filtered through five layers of *Whatman* glass fiber filters. The filtrate was then lyophilized overnight. Analysis was mainly done by qNMR occasionally supported by IR spectroscopy. The analysis of branched high molecular weight dextran became more challenging due to a broader NMR signal. This led to less accurate integration.

**Table 1 Aerobic oxidation of dextran in the PolyBLOCK® reactor with palladium neocuproine in aq. solutions.**

| M Substr. [kDa] | co-solvent [v%] | Cat. loading [mol%] | n(Glc units) [mmol] | n(cat.) [mmol] | [rel.] I(SM) | [rel.] I(P) | TON | Oxidation degree [%] |
|---|---|---|---|---|---|---|---|---|
| 6 | 20 | 0.5 | 12.335 | 0.053 | 2.90 | 1 | 30/ 20 h | 26 |
| 6 | 20 | 0.5 | 9.251 | 0.040 | 1.73 | 1 | 12/ 20 h* | 37 |
| 70 | 20 | 0.5 | 12.335 | 0.057 | 15.62 | 1 | 6/ 20 h | 6 |
| 70 | 20 | 0.5 | 12.335 | 0.057 | 11.86 | 1 | 2/ 20 h* | 8 |
| 6 | 20 | 0.23 | 12.335 | 0.029 | 4.62 | 1 | 38/ 20 h | 17 |
| 6 | 20 | 0.31 | 12.335 | 0.038 | 4.33 | 1 | 36/ 20 h | 21 |

Conditions: Dextran in $H_2O$/MeCN (0.5 M), palladium(II) neocuproine triflate as catalyst (1 mol% of Pd), 1 atm $O_2$, at 45 °C with a stirring speed of 600 rpm for 20 h. *2$^{nd}$ addition of catalyst with filtration after 20 h, and then stirring for another 20 h.

[(Neocuproine)PdOAc]$_2$OTf$_2$)] was prepared following the literature procedure. Spectral data corresponded with literature.[3]

Despite the optimizations that were done on the oxidation of dextran using palladium(II) neocuproine triflate as catalyst, the activity of the catalyst decreased over time due to the two ways of degradation described earlier by Waymouth *et al*. (Fig. 4) and was, therefore, removed and not reused.

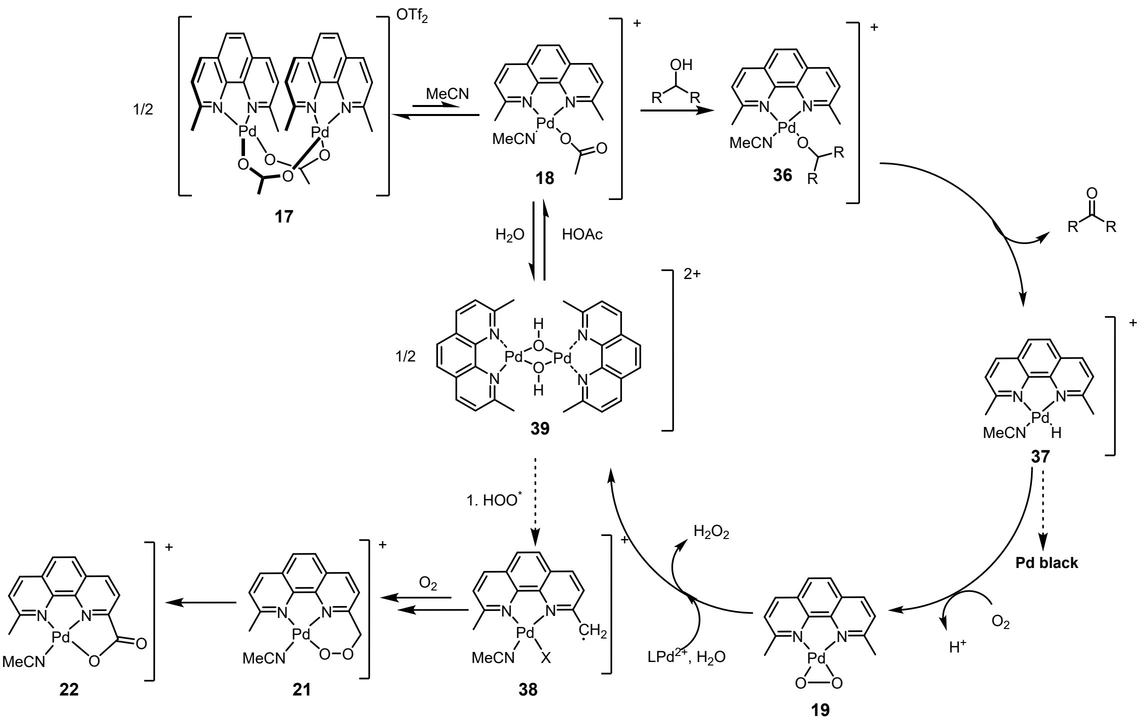


**Fig. 5 Full mechanistic cycle of the complex during aerobic oxidation with two ways of catalyst degradation. Adapted from Waymouth *et al.*[3]**

# 3 NMR and FTIR spectra of (keto-)dextran

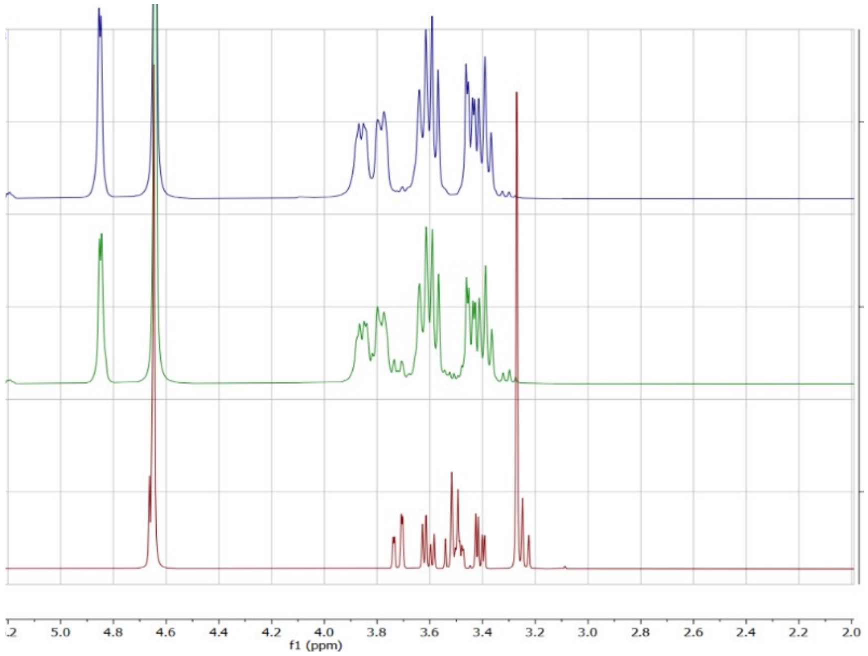


**Fig. 6 qNMR spectra in $D_2O$ of red: α-methyl-ᴅ-glucoside, green: native linear low molecular weight dextran (6 kDa), and blue: native high molecular weight branched dextran (70 kDa).**

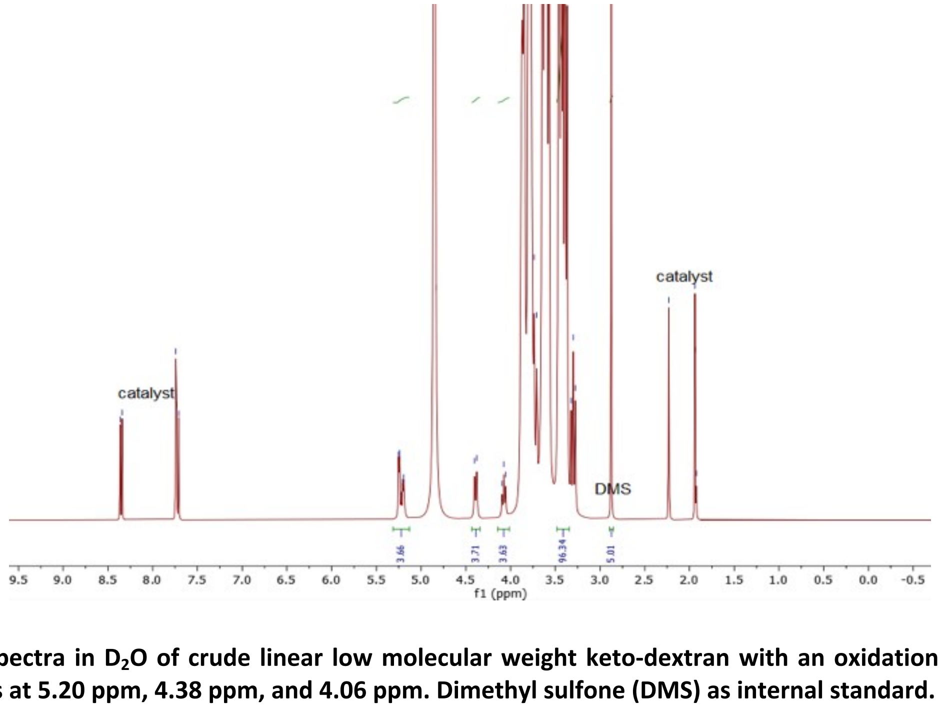


**Fig. 7 qNMR spectra in $D_2O$ of crude linear low molecular weight keto-dextran with an oxidation grade of ~4%. Product signals at 5.20 ppm, 4.38 ppm, and 4.06 ppm. Dimethyl sulfone (DMS) as internal standard.**

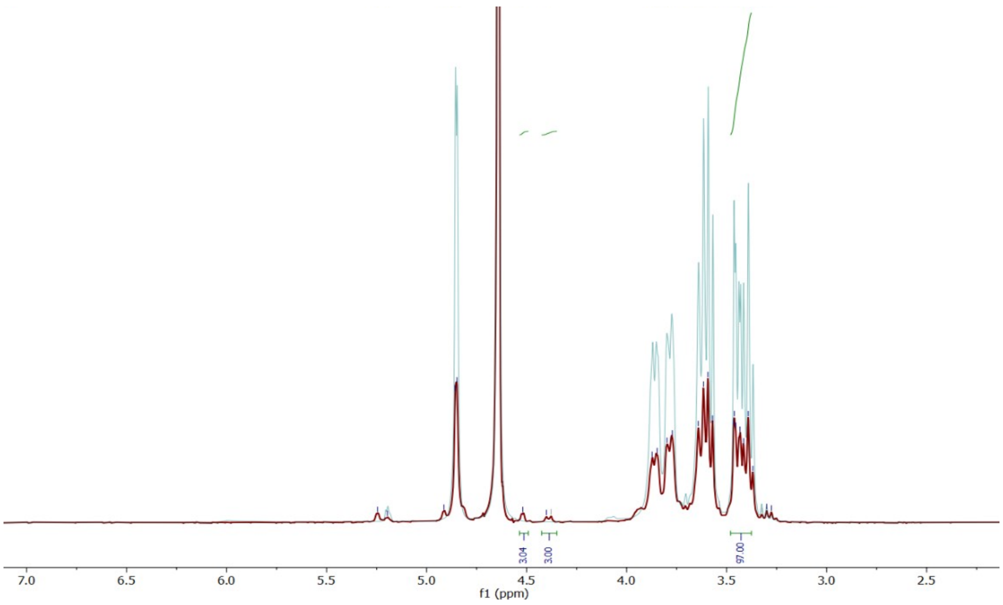


**Fig. 8 qNMR spectra red: native branched dextran with a molecular weight of 70 kDa, and petrol: branched keto-dextran with a molecular mass of 70 kDa and an oxidation degree of 3%.**

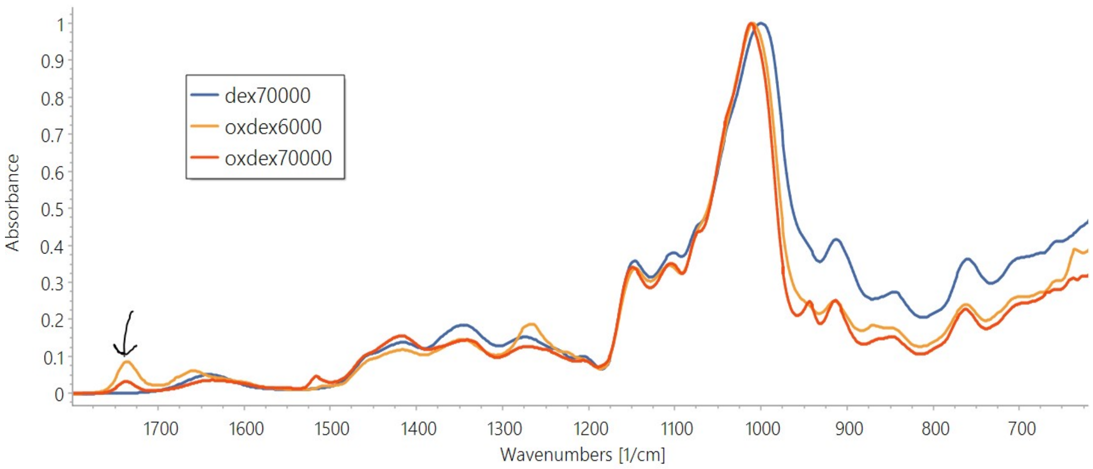


**Fig. 9 FTIR spectra of solid native branched dextran (70 kDa), oxidized keto-dextran (6 kDa & 70 kDa).**

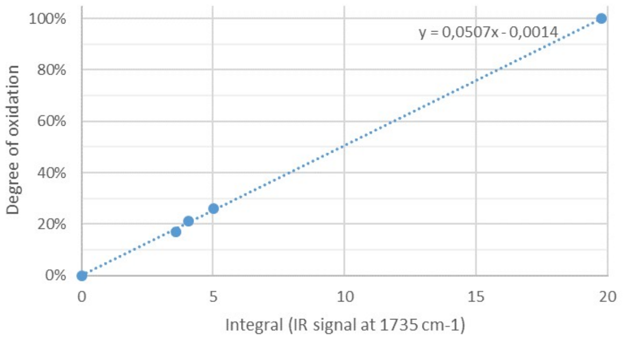


**Fig. 10 Calibration curve for oxidation degree of dextran.**

**Table 2 Comparing the degree of oxidation of different keto-saccharides analyzed by qNMR and IR.**

| Substrate | IR Integral (at 1735 cm-1) | Degree of oxidation (IR) | Degree of oxidation (qNMR) |
|---|---|---|---|
| Linear dextran | 0 | 0% | 0% |
| Linear keto-dextran | 3.608 | 18% | 17% |
| Linear keto-dextran | 4.078 | 21% | 21% |
| Linear keto-dextran | 5.039 | 26% | 26% |
| Keto-glucoside | 19.76 | 101% | 100% |
| Branched keto-dex | 1.21 | 6% | 6% |
| Branched keto-dex | 1.61 | 8% | 8% |

Equation of the calibration curve for calculating the oxidation degree with IR: y=0,0512x-0,0014.

Oxidation of positions other than the C3-OH in the glucose monomer unit was not observed in the NMR spectra but potentially occurred at very low levels.

## 4 Fragmentation studies of keto-dextran

To a solution of 6 kDa linear keto-dextran (400 mg, 0.067 mmol)) with an oxidation degree of 10% in 8 mL water, $K_2CO_3$ (90 mg, 0.651 mmol, 9.77 equiv.) was added. The reaction mixture was heated to 40 °C and stirred overnight. Subsequently, 0.5 mL of the solution was filtered by cut-off centrifugal filters (Amicon® Ultra-0.5 Centrifugal Filter for 5 kDa, 3 kDa, and 1 kDa). The filtrate was lyophilized, weighted (25 mg ± 0.5 mg remaining) and afterwards analyzed by qNMR in $D_2O$ as solvent.

## 5 Droplet coatings of keto-dextrans with ADH and their analysis by IR spectroscopy

The keto-dextrans were stored at -20 °C and taken as bio-based binders for cross-linking experiments. The solid content of the aqueous solution with the dextrans was 10 w% in order to match the rheological behavior of the commercial binder.[4] The degree of oxidation varied up to 37%.

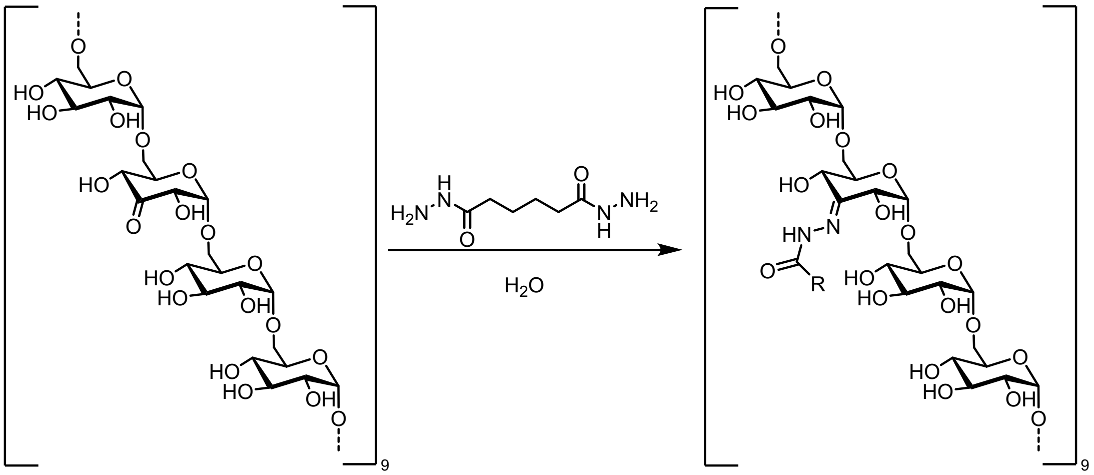


**Fig. 11 Cross-linking reaction of linear low molecular weight dextran with a degree of oxidation of 40% with ADH as crosslinker in water.**

General procedure of droplet coatings (if not mentioned otherwise)

Several dextrans (each 100 mg) were dissolved in water (each 0.9 mL, 10 w% solid content) and ADH (0.5 equiv. per keto-moiety) was added as a solid. The reaction mixture was stirred for 30 min at r.t. Afterwards, 4 times 50 µL was applied as a droplet on a glass microscopy plate.

Water resistance

After drying for 24 h at r.t., the water resistance of the droplet coatings was tested by putting the coated glass microscopy plates in a standing position and pouring 10 mL over each droplet. A graphical representation of smearing patterns can be found in **Fig. 10**.

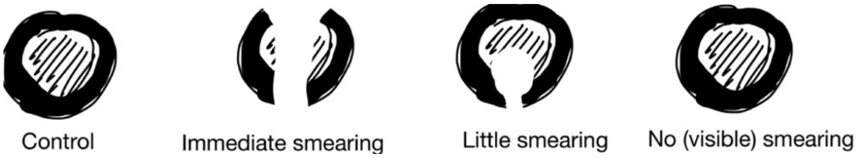


**Fig. 12 Graphical representation of smearing patterns.**

**Table 3 Summary of the water resistance tests of droplet coatings with different dextran polymers and ADH as crosslinker.**

| **Conditions** | **Linear dextran 0% ox. degree** | **Keto-dextran 5-10% ox. degree** | **Keto-dextran 17-21% ox. degree** | **Keto-dextran 26-37% ox. degree** |
|---|---|---|---|---|
| 0.5 equiv. ADH No AcOH | Immediate smearing | Immediate smearing | Little smearing (inside) | No smearing but swelling |

Conditions: Dextran in water (10 w% solid content) with 0.5 equiv. ADH per keto-moiety. For native dextran, the same amount of ADH was added as for an oxidation degree of 10%. Stirring for 30 min at r.t. of the reaction mixture before applying as droplet coating on glass microscopy plates and drying for 24 h at r.t.

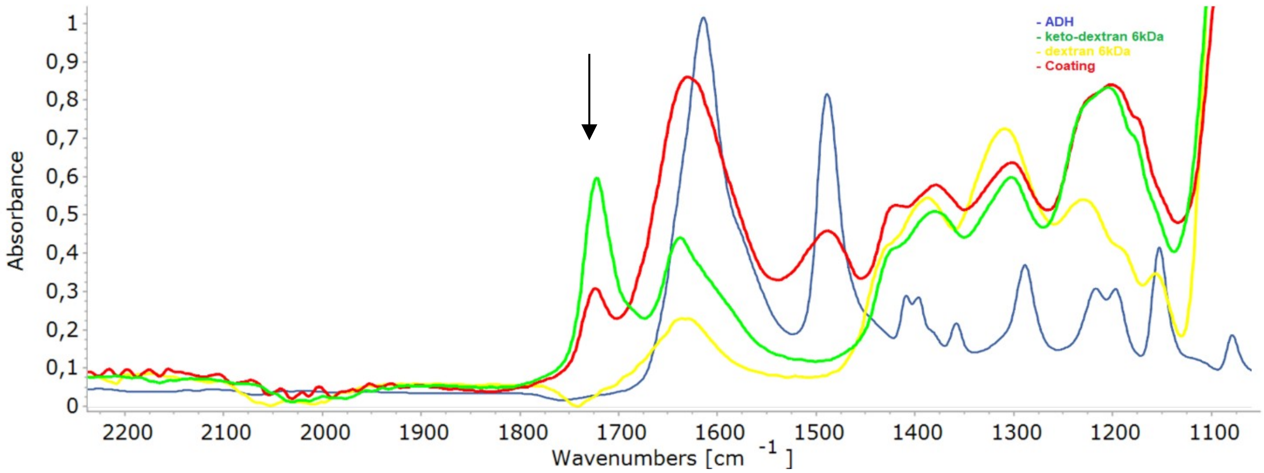


**Fig. 13 FTIR spectra of solid linear native dextran, of solid linear dextran with an oxidation degree of 21%, and of solid linear dextran with an oxidation degree of 21% with ADH ("coating"), as well as, only solid ADH as reference spectra. Y-axis was normalized at 1000 cm$^{-1}$.**

# 6 Bar coatings of keto-dextrans with a commercial keto-functionalised Joncryl® binder and ADH

Composition of mixtures

The BASF keto-functionalized Joncryl® binder normally is a self-crosslinking acrylic emulsion with ADH already added. It is a binder for water-based inks used for surface printing on film substrates. In order to analyze the co-cross-linking behavior with the oxidized dextran, this time the binder was prepared without the addition of ADH, the latter was added later in the desired amounts. The commercial binder consists of 5% diacetone acrylamide (DAAM) which is, hence, also the degree of functionalization (ketones).

The binder blends were prepared by mixing Joncryl® (emulsion with a solid content of 43%) with the bio-polymer (solution with a solid content of 15%) in various ratios, and water, resulting in an overall binder's solid content of ~30 w%. After stirring this mixture for 15 min at r.t. (1000 rpm with an overhead agitator), ADH (as a solution with 8 w% solids) was added and stirring was continued for another 30 min at r.t. Subsequently, the emulsion was distributed over two vials and to one vial a blue pigment was added. Both emulsions were then taken for bar-coatings on an unsealed (semi-porous) Leneta N2A-test chart whose surface simulates wood or unsealed wallboard.

**Table 4. Composition of coatings of cross-linking with keto-functionalized binder Joncryl®, bio-polymer, and ADH.**

| Amount bio | 0% | ~5w% | ~10w% | ~15w% | ~20w% |
|---|---|---|---|---|---|
| **m (binder)** | 25 | 22.5 | 20 | 17.5 | 15 |
| **amount solids** | 0.43 | 0.43 | 0.43 | 0.43 | 0.43 |
| **m (solids)** | 10.75 | 9.675 | 8.6 | 7.525 | 6.45 |
| **m (water in binder)** | 14.25 | 12.825 | 11.4 | 9.975 | 8.55 |

| | | | | | |
|---|---|---|---|---|---|
| **m (bio)** | 0 | 2.5 | 5 | 7.5 | 10 |
| **amount solids** | 0 | 0.15 | 0.15 | 0.15 | 0.15 |
| **m (solid)** | 0 | 0.375 | 0.75 | 1.125 | 1.5 |
| **m (water in solution)** | 0 | 2.125 | 4.25 | 6.375 | 8.5 |
| **m (ADH)** | 3.58 | 3.54 | 3.5 | 3.46 | 3.43 |
| **amount solids** | 0.08 | 0.08 | 0.08 | 0.08 | 0.08 |
| **m (solid ADH)** | 0.286 | 0.283 | 0.28 | 0.277 | 0.274 |
| **m (water in ADH solution)** | 3.294 | 3.257 | 3.22 | 3.183 | 3.156 |
| **m (extra water)** | 1.42 | 1.46 | 1.5 | 1.54 | 1.57 |
| **m(total)** | 30 | 30 | 30 | 30 | 30 |
| **% bio (binder)** | 0 | 4 | 9 | 15 | 23 |
| **% dextran (solids in total)** | 0 | 4 | 8 | 13 | 18 |
| **solid content in total** | 37 | 34 | 32 | 30 | 27 |

Bio-polymer: Maltodextrin, linear dextran with a molecular weight of 6 kDa, linear keto-dextran with an oxidation degree of 10%, 21% or 27%. Addition: ADH was not used to calculate the bio-content because it can come from different sources (bio-based possible).[5]

A paint applicator from RK print-coat instrument Ltd. England was used (**Fig. 12 right**) for applying the bar coatings. ~1.5 mL of the clean mixture as well as the pigmented mixture were pipetted next to each other on the Leneta test chart (**Fig. 12 left**). The bar speed was set to 8. The chosen film thickness was 12 µm. The coatings were directly dried at 60 °C for 1 min and then at r.t. for 24 h. The wire wound bar was cleaned after every run with tap water and a brush.

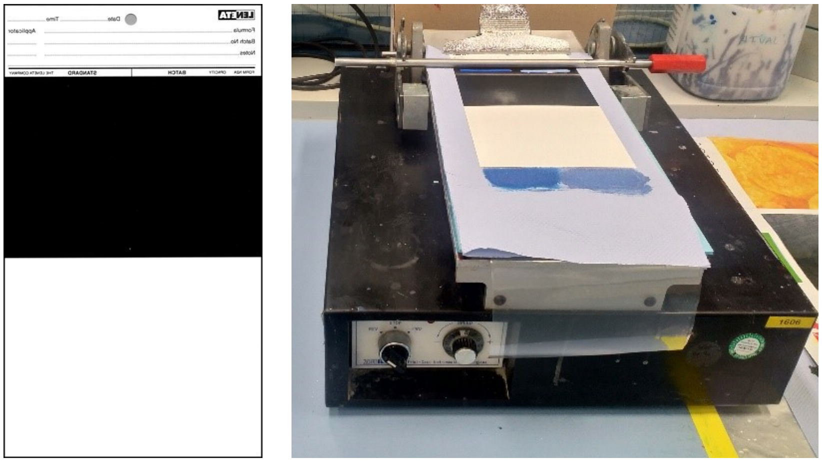

**Fig. 14 Left: Leneta N2A unsealed test chart; left: Set-up of bar coating experiments at BASF using a paint applicator from RK print-coat instrument Ltd. England.**

## 7 Photo evidence of gelation

Upon mixing Joncryl® with the keto-dextran and ADH, several of the blends showed immediate gelation, evidenced by the formation of a precipitate on the inside wall of the vial (**Fig. 13**). It was not possible to redissolve the formed solids by adding more water, which was visible in particular on the inside wall of the vial.

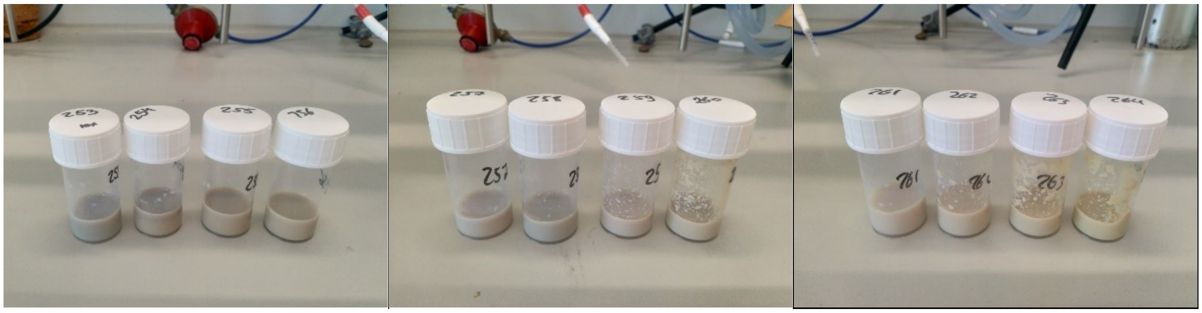

**Fig. 15 All three photos from left to right 5 w%, 10 w%, 15 w%, and 20 w% of keto-dextran with Joncryl® binder and ADH in water. Left photo with keto-dextran of an oxidation degree of 10%; middle photo with keto-dextran of an oxidation degree of 11%; and right photo with keto-dextran of an oxidation degree of 27%.**

## 8 Water resistance tests with photo evidence

After drying of the coatings, 4 water droplets (~100 µL) were pipetted on each test chart (one per section). After 1 min, a wet piece of felt was taken and rubbed 30 times on the soaked areas. Then the spots were examined on their colour and gloss (by eye) to evaluate the water resistance. Moreover, a water-soaked towel was put on each section for 60 min in order to test the long-term water resistance. Afterwards, a wet piece of felt was taken again and rubbed 30 times on the soaked areas.

The upper half of the test chart has a black background whereas the lower half is white. While the black background is convenient to look at changes in the gloss of the coating, the white background comes in handier to investigate the water resistance by looking at the possible disappearance of the pigmented (blue) coating.

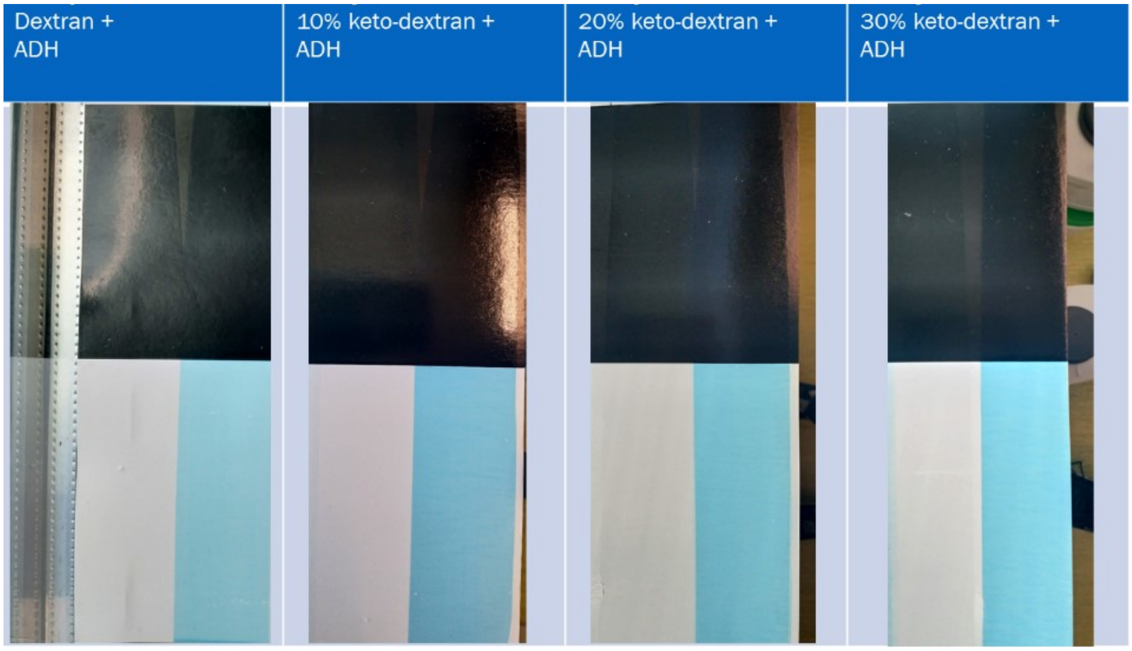


**Fig. 16 Final result of water resistance tests with 5w% bio-polymer and 95 w% Joncryl® binder and stoichiometric amounts of ADH. Degree of oxidation of keto-dextrans in title.**

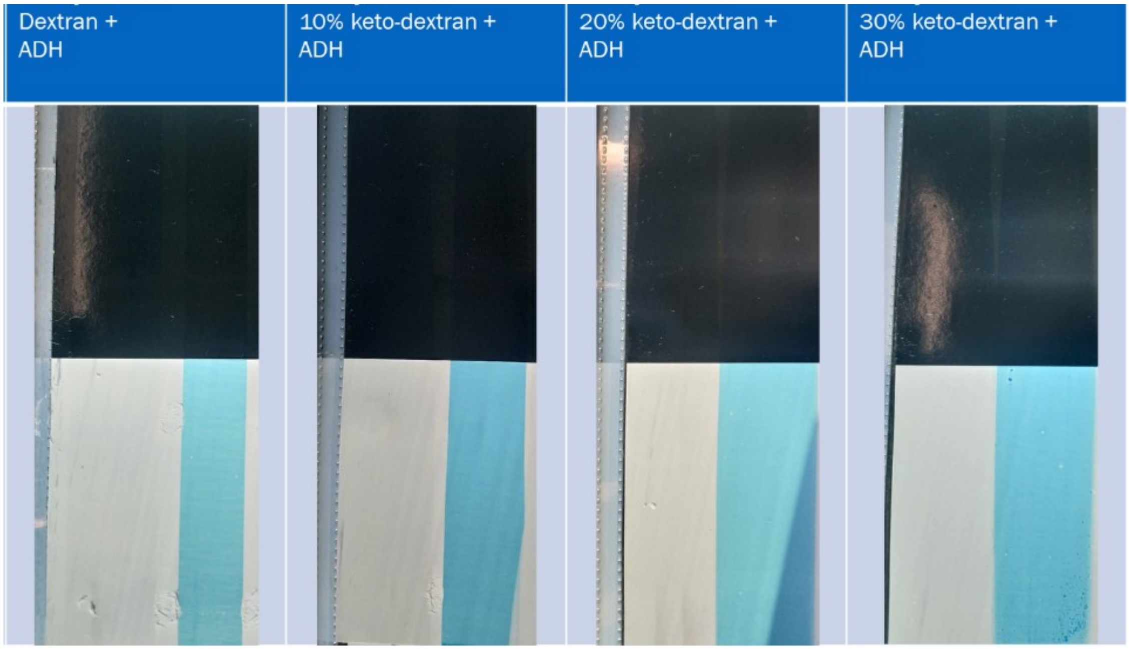


**Fig. 17 Final result of water resistance tests with 10w% bio-polymer and 90 w% Joncryl® binder and stoichiometric amounts of ADH. Degree of oxidation of keto-dextrans in title.**

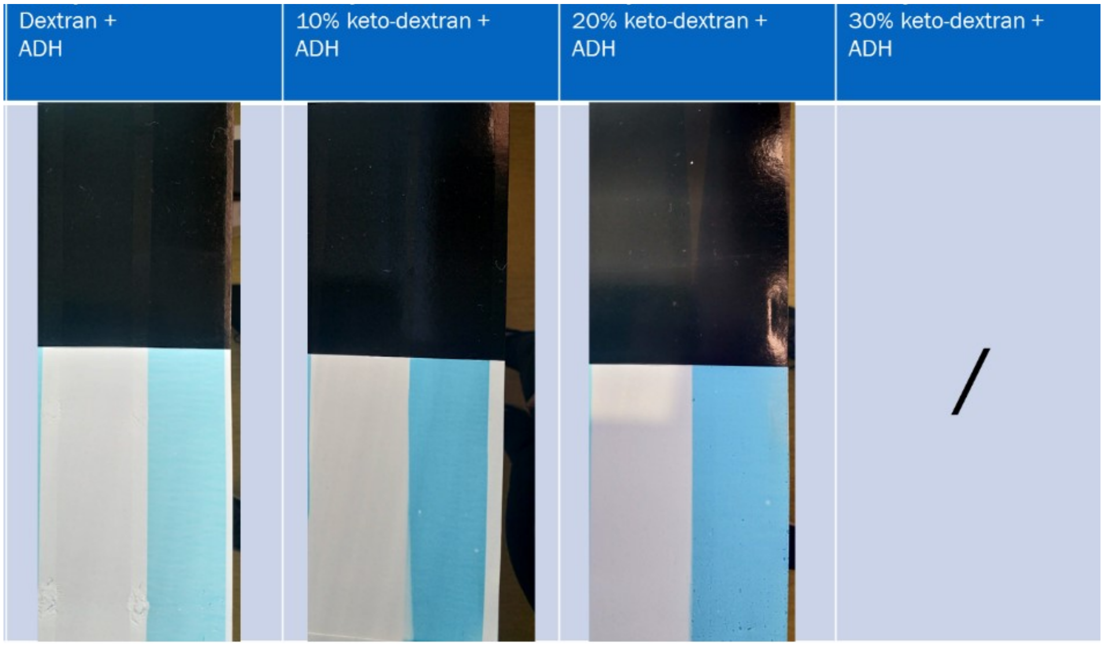


**Fig. 18 Final result of water resistance tests with 15w% bio-polymer and 85 w% Joncryl® binder and stoichiometric amounts of ADH. Degree of oxidation of keto-dextrans in title.**

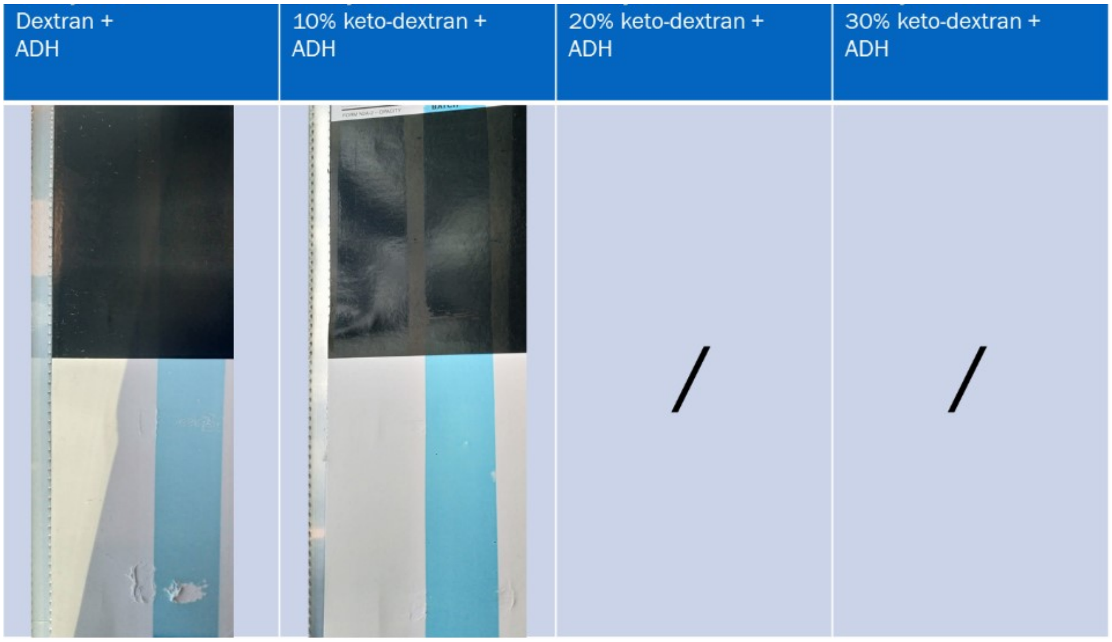


**Fig. 19 Final result of water resistance tests with 20w% bio-polymer and 80 w% Joncryl® binder and stoichiometric amounts of ADH. Degree of oxidation of keto-dextrans in title.**

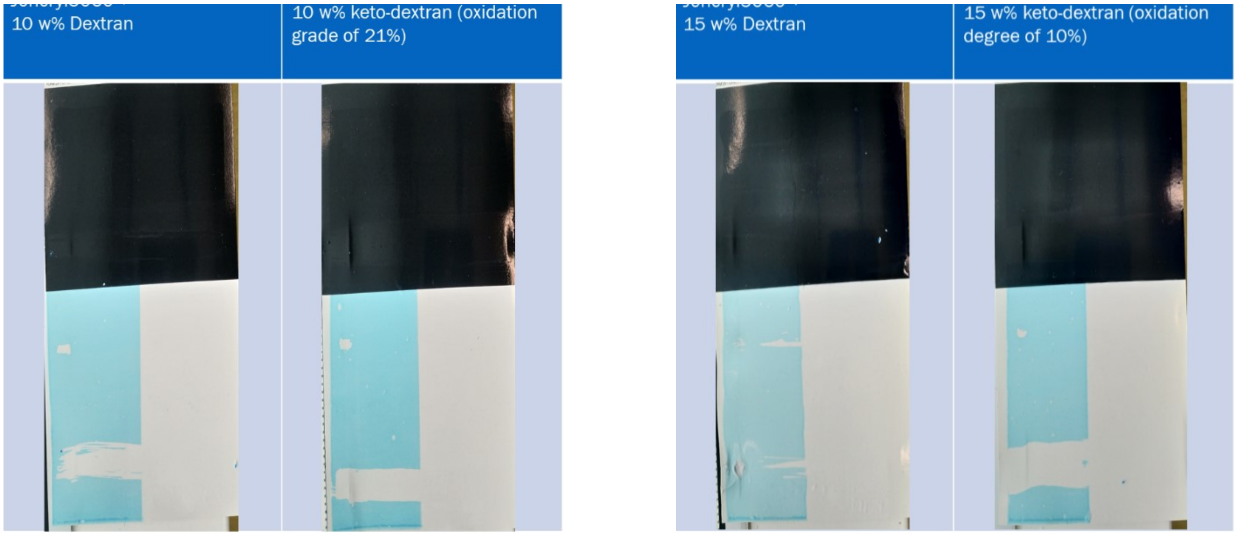


**Fig. 20 Final result of reference water resistance tests with 10 w% or 15 w% bio-polymer without ADH on *Leneta* 2A sealed test charts. Degree of oxidation of keto-dextrans in title.**

## 9 References


1. R. L. Sidebotham, in *Advances in Carbohydrate Chemistry and Biochemistry*, eds. R. S. Tipson and D. Horton, Academic Press, 1974, vol. 30, pp. 371-444.
2. N. N. H. M. Eisink, J. Lohse, M. D. Witte and A. J. Minnaard, *Org Biomol Chem*, 2016, **14**, 4859-4864.
3. W. C. Ho, K. Chung, A. J. Ingram and R. M. Waymouth, *J. Am. Chem. Soc.*, 2018, **140**, 748-757.
4. V. Kokol, *Carbohydrate polymers*, 2002, **50**, 227-236.
5. Y. S. Jang, B. Kim, J. H. Shin, Y. J. Choi, S. Choi, C. W. Song, J. Lee, H. G. Park and S. Y. Lee, *Biotechnology and bioengineering*, 2012, **109**, 2437-2459.
